# Cost and effects of integrated care: a systematic literature review and meta-analysis

**DOI:** 10.1007/s10198-020-01217-5

**Published:** 2020-07-06

**Authors:** Stephen Rocks, Daniela Berntson, Alejandro Gil-Salmerón, Mudathira Kadu, Nieves Ehrenberg, Viktoria Stein, Apostolos Tsiachristas

**Affiliations:** 1 Health Economics Research Centre, Nuffield Department of Population Health, University of Oxford, Oxford, UK; 2 Polibienestar Research Institute, Universitat de València, Valencia, Spain; 3 Institute of Health Policy, Management and Evaluation, University of Toronto, Toronto, ON Canada; 4 International Foundation for Integrated Care, Oxford, UK

**Keywords:** Cost-effectiveness, Economic evaluation, Integrated care; meta-analysis

## Abstract

**Background:**

Health and care services are becoming increasingly strained and healthcare authorities worldwide are investing in integrated care in the hope of delivering higher-quality services while containing costs. The cost-effectiveness of integrated care, however, remains unclear. This systematic review and meta-analysis aims to appraise current economic evaluations of integrated care and assesses the impact on outcomes and costs.

**Methods:**

CINAHL, DARE, EMBASE, Medline/PubMed, NHS EED, OECD Library, Scopus, Web of Science, and WHOLIS databases from inception to 31 December 2019 were searched to identify studies assessing the cost-effectiveness of integrated care. Study quality was assessed using an adapted CHEERS checklist and used as weight in a random-effects meta-analysis to estimate mean cost and mean outcomes of integrated care.

**Results:**

Selected studies achieved a relatively low average quality score of 65.0% (± 18.7%). Overall meta-analyses from 34 studies showed a significant decrease in costs (0.94; CI 0.90–0.99) and a statistically significant improvement in outcomes (1.06; CI 1.05–1.08) associated with integrated care compared to the control. There is substantial heterogeneity in both costs and outcomes across subgroups. Results were significant in studies lasting over 12 months (12 studies), with both a decrease in cost (0.87; CI 0.80–0.94) and improvement in outcomes (1.15; 95% CI 1.11–1.18) for integrated care interventions; whereas, these associations were not significant in studies with follow-up less than a year.

**Conclusion:**

Our findings suggest that integrated care is likely to reduce cost and improve outcome. However, existing evidence varies largely and is of moderate quality. Future economic evaluation should target methodological issues to aid policy decisions with more robust evidence on the cost-effectiveness of integrated care.

**Electronic supplementary material:**

The online version of this article (10.1007/s10198-020-01217-5) contains supplementary material, which is available to authorized users.

## Introduction

Governments across high-income countries are challenged to contain the relentless increase in health expenditure, which is partly driven by ageing populations and an associated increase in the prevalence of chronic disease [1]. This challenge is increasingly concerning as many health systems have become highly specialised, fragmented and poorly set to manage the growing burden of multimorbidity [2]. Increasing efficiency in care delivery by integrating health services has been proposed as a solution to healthcare budget issues [3–5]. Integrated care is an umbrella term that encompasses a diverse set of methods and models that facilitate improvement in patient experience through enhanced coordination and continuity of care [6, 7]. As such, integrated care covers a wide range of treatment plans and organizational models, does not discriminate between diseases or target populations, and has varied consequences across different treatment areas [8]. The Triple Aim of integrated care is to improve population health, enhance user experience with care and reduce growing healthcare expenditure [9–12].

Despite pressure to assess the cost-effectiveness of integrated care interventions to appropriately inform decision makers of the potential financial benefits of moving towards new models of care [13], the evidence from economic evaluations has thus far been inconclusive [14–16]. This is widely attributed to the lack of reliable evidence summarized by Nolte and colleagues (2014) who identified the “quality of existing economic evaluations as the main impediment to arriving at robust evidence to inform decision making” [17]. Indeed, a recent systematic literature review showed that economic evaluations in integrated care had on average poor-to-moderate methodological quality [18]. In addition, the unclear definition of what constitutes integrated care and the large variation in the models of integration within and across geographic areas poses a challenge to the reliability and replicability of evaluation studies [19].

Currently, there is a dearth of meta-analyses of economic evaluations that compare integrated care models with conventional care; this evidence focuses on single conditions and certain models of integrated care [14, 20, 21]. In addition to the limited span of evidence, the overall impact of integrated care on costs and outcomes is still unclear. This paper aims to provide an up-to-date review of economic evaluations in integrated care and perform a meta-analysis to summarize the impact of integrated care on costs and outcomes.

## Methods

### Search strategy

We followed the search strategy of a recently published review of economic evaluations in integrated care that assessed their methodological quality [18]. For this updated literature review, we searched for eligible studies from inception to 31st December 2019 in the following databases: CINAHL, Database of Abstracts of Reviews of Effects (DARE), EMBASE, Medline/PubMed, the NHS Economic Evaluation Database (NHS EED), OECD Library, Scopus, Web of Science, and the World Health Organization library and information networks for knowledge database (WHOLIS).

Terms relating distinctly to the broad concepts of “integrated care” and “economic evaluation” were identified in relevant journals (International Journal of Integrated Care) and previous systematic reviews that reported on the cost-effectiveness of integrated care [14]. Our search was also informed by terms used in the Integrated Care Search tool developed by the International Foundation for Integrated Care [22]. Frequently used expressions were then compiled to form the list of search terms, shown in Supplementary material Figure S1. Additionally, reference lists featured in key publications [17] (including systematic reviews, opinion pieces and editorials on integrated care) were hand-searched to identify any relevant articles that were otherwise missed [23, 24].

### Selection process and eligibility criteria

All study titles and abstracts were added in an online citation manager tool, Mendeley. Study selection occurred in a two-step process based on the pre-set inclusion and exclusion criteria detailed below. First, titles and abstracts were screened by DB or AGS, and second, full text of selected studies were screened for final inclusion. To ensure consistency between the reviewers, AT screened the titles, abstracts, and full texts of 10% of the articles screened and selected by the other reviewers [25, 26]. The target for inter-reviewer agreement was 95%. When there was uncertainty over whether to exclude an article based upon understanding of its title and abstract the full text was read and its inclusion was discussed between DB, AGS and AT until a decision was made.

Inclusion criteria:

1. Articles describing the implementation, execution or evaluation of interventions or programs based on the most frequently used definition of integrated care: “funding, administrative, organisational, service delivery and clinical interventions designed to create connectivity, alignment and collaboration within and/or between the cure and care sectors” [27].
2. Articles including empirical economic evaluations as defined by Drummond and colleagues: “the comparative analysis, measurement, valuing and identification of alternative courses of actions in terms of their cost and consequences” [28]*.*

Exclusion criteria:

1. Articles published in languages other than English.
2. Articles solely describing the concept of integrated care or the rationale behind its implementation without reporting on an actual intervention or practice.
3. Systematic reviews, dissertations, conference proceedings, opinion pieces, editorials and conference abstracts on the subject of integrated care.

### Data abstraction and quality assessment

Elements from the PICO model [29] and abstraction templates designed by Boland and colleagues [20] were adapted for the purpose of qualitative data abstraction; data included sample sizes, study origin, follow-up period, study design and objectives, patient and intervention characteristics, whilst also describing the perspective from which the economic evaluation was taken (Supplementary material Table S1).

A second template, designed as a checklist, was developed as a tool to assess the overall quality of each study and their economic evaluations. The Consolidated Health Economic Evaluation Reporting Standards (CHEERS) [30] and the health technology assessment of disease management programs (HTA-DM) [31] were consolidated into a list of 30 items upon which a binary scoring system acted to assess study strengths and weaknesses (Supplementary material Table S2). Each study was given a quality score to assess overall study quality. The quality score was a proportion with a maximum of 100% (highest possible quality), where the numerator is the sum of the binary scores (maximum of 30) divided by the denominator (30 minus any not-applicable scores).

A final template was designed to collate both qualitative and quantitative data regarding the outcome measures of treatment cost and effectiveness (see Supplementary material Tables S3 and S4). Principal information extracted included: currency; cost categories included in total cost; mean total cost of intervention and control; mean cost ratio; ICER, if included; measurement of quality of life; mean quality of life score; and mean quality of life ratio.

All templates were initially trialled on the data abstraction of four studies before use on all selected studies.

### Meta-analysis and statistical approaches

Studies found to contain insufficient cost or outcome data during data extraction were excluded from the meta-analysis. Cost and outcome data extracted from the same studies were analyzed separately.

In the case of cost analyses, the ratio of final costs associated with the intervention and control group was calculated for each study. The use of a cost ratios annuls differences in measurement across studies, which means currency conversions, inflation compensations, and standardization of varying cost scales were not necessary. The majority of studies did not report any standard deviation nor standard error of the reported costs and outcomes; therefore, we followed similar examples in the literature and used the study quality scores generated to weigh studies in the meta-analysis [32, 33]. In this way, higher-quality studies were given greater weight and lower-quality, and therefore less reliable, studies less weight.

For the assessment of care effectiveness, the ratio of mean effectiveness scores associated with the intervention and control group was calculated. The use of a ratio helps account for disparities in the measures used to assess effectiveness of care in individual studies, facilitating the pooling and comparison of their data [34]. In the case of a lower effect score translating to a better clinical outcome, the effect ratio was inverted to reflect the appropriate change in outcome.

For observational studies that did not control for confounding factors at baseline, additional adjustments were undertaken to compensate for baseline differences when assessing costs (Eq. 1) and effects (Eq. 2).

1 $$\frac{Final mean cost of intervention}{Final mean cost of control} \times \frac{Baseline mean cost of control}{Baseline mean cost of intervention}$$

2 $$\frac{Final mean effect of intervention}{Final mean effect of control} \times \frac{Baseline mean effect of control}{Baseline mean effect of intervention}$$

Stata12.1 SE (StataCorp, Tx) was used when undertaking analyses. To calculate a weighted average ratio of mean cost and outcome, the data were pooled using a random-effects meta-analysis model based on the DerSimonian–Laird method [35]. The variety in patient characteristics, and hence true effect size, across studies justified the use of random-effects meta-analysis. Heterogeneity in the results was then illustrated by forest plots grouped into study- (1) design, (2) duration, (3) region and (4) type of integrated care intervention.

## Results

### Search results

**Fig. 1:**
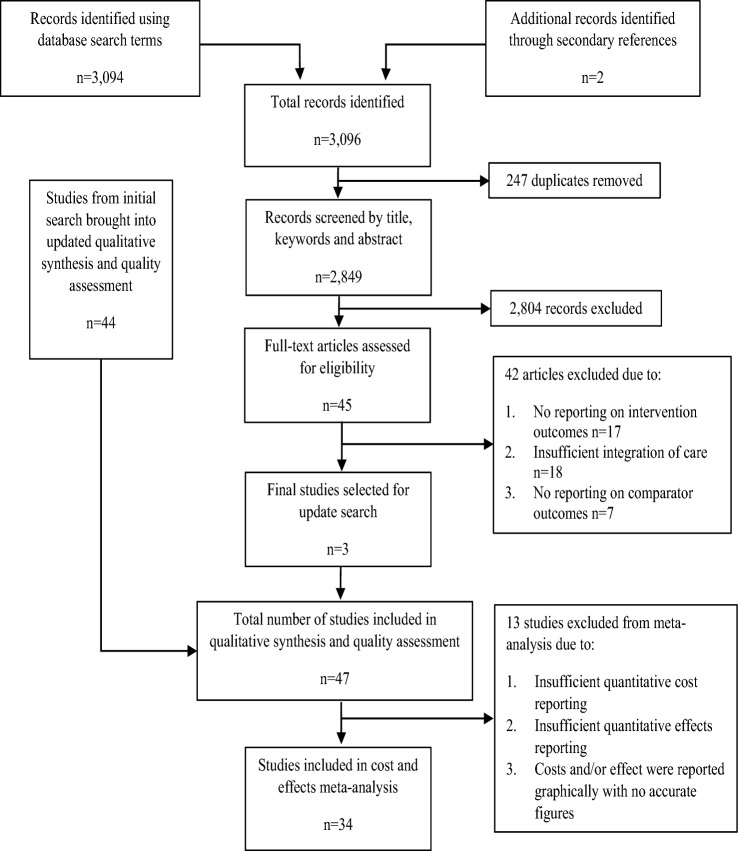
Updated flow chart of study selection

The initial review included 44 scientific articles and the detail of the selection process is described elsewhere [18]. The review update yielded a total of 3094 articles, with two further articles [23, 24] identified from subsequent citation screening of relevant reference lists. Following removal of duplicates and screening of title and abstract, 45 eligible studies remained for full-text examination. Finally, three studies [36–38] from the update search were considered to follow all pre-set inclusion and exclusion criteria and included in the final quality assessment and meta-analysis. Combining the initial and update reviews, 47 studies were brought forward for the quality assessment and data extraction. Of those, 13 studies were excluded from the meta-analysis due to inadequate cost and outcome reporting [39–51]. The reported costs and outcomes of 34 studies were used in the meta-analyses [24, 36–38, 52–80]. The adapted PRISMA flowchart of the review update is presented in Fig. 1 and follows Cochrane guidance for updated reviews [81].

### Study description

There were sixteen (34%) randomized control trials (RCT), twelve (26%) cluster RCTs, ten (21%) pre-post cohort studies, eight (17%) cohort studies and one (2%) cross-sectional study included (Supplementary material Table S1). Twenty-four (51%) of these studies originated from Europe, whilst 16 (34%) studies were conducted in North America, three (6%) in Africa, two (4%) in Asia, and two (4%) in Australia. Study observation period varied from three to 36 months, excluding one cross-sectional study that assessed a large population [54]*.* Thirty-one of the selected studies were conducted from the healthcare payer perspective (66%), whilst the remaining 16 adopted a societal perspective (33%). Twenty-seven studies (57%) had a sample size smaller than 500 patients.

Different types of integrated care strategies were identified and grouped in integrated case management interventions (*n* = 10, 21%), integrated care teams (*n* = 9, 19%), coordination between different services (*n* = 7, 15%), integrated care pathways (*n* = 4, 9%) and integrated care programs based on disease management (*n* = 4, 9%). The majority of the studies used care as usual as a comparison group (98%), just one study estimated the cost-effectiveness of different levels of disease management intensity [24].

Principal outcomes extracted to assess effectiveness were quality of life (QOL) and quality-adjusted life-years (QALY), although several studies opted to measure mortality rates [52, 61, 73, 74] or other clinical performance scores [54, 57, 65, 68, 72] (Supplementary material Table S4). Economic results were largely reported as mean costs of the implemented integrated care program in comparison to the equivalent costs borne by the control group. Three studies [52, 55, 64], however, reported the costs of the control and intervention groups after both a baseline period of 3 months and after implementation of the intervention programme. Similar baseline reporting practice was also undertaken in six studies with regards to the measuring of effectiveness [36, 64, 68, 69, 71, 74]. In these cases, the final mean costs or outcomes of each group were adjusted (using Eq. 1 and 2) for their differing baseline values before entering the meta-analysis.

### Quality assessment

The quality score of the 47 selected studies varied from 27.6% [43] to 96.7% [62] (Supplementary material Table S2) with a mean value of 65.6% (± 18.5). All studies had clear a description of the intervention strategy and outcomes measured. All but four studies featured an intervention and control group; the remaining studies instead used a pre-post comparison without a control group [40, 44, 63, 74]. Study populations were randomly allocated to either the control or intervention group in 60% of cases. Whilst most of the remaining 40% of studies used tools such as propensity score matching to adjust for differences between the intervention and control group, several observational studies were subject to selection bias and made no attempt to adjust for confounding factors at baseline. Although most studies defined their inclusion and exclusion criteria (87%), 26% did not provide information about any dropouts sustained during the study period. Furthermore, despite a large proportion (85%) of the selected studies describing relevant aspects of the systems in which the intervention took place, only 36% of studies proceeded to take measures to avoid co-intervention or contamination within said system.

Descriptions of both outcome measures and sources of resource utilization were clear in 100% and 91% of studies respectively. The reporting of costs, however, was less promising and often exclusively covered direct medical costs of the intervention and control, while only 38% of selected studies included development and implementation costs of the intervention programme. In addition, only 24% of studies with time horizons over 12 months performed cost discounting [49, 58, 66, 75]. Nevertheless, cost and utilization across both social and health sectors were measured in 70% of selected studies.

Statistical analyses performed in the studies varied substantially in quality. Although relevant information of all parameters was reported in the vast majority of studies (91%), 62% dealt with missed observations [82] and 70% appropriately handled skewed data [83]. Variability is inherent due to the nature of these study populations, particularly those providing interventions to larger sample sizes [46, 47, 54, 61, 64, 72–75, 84]. Despite this irregularity in baseline characteristics, most studies (51%) did not perform sub-group analysis to examine heterogeneity of the results. Cost-effectiveness was analyzed using the incremental cost-effectiveness ratio (ICER) method in 57% of the studies, while only 11% opted for either net-monetary or net-health benefit analysis [70, 72, 76].

### Reported costs and effects

Reported costs and outcomes are summarized in Supplementary material Tables S3 and S4. Studies included different types of costs in their total cost sum, including the following cost categories: inpatient, outpatient, development, implementation, societal, travel and productivity losses. Similarly, when reporting relative effectiveness, a variety of outcome measures were used across the studies. The outcomes observed included: quality of life (e.g., SF-12, EQ-5D, WHOQOLBRIEF), quality-adjusted life-years (QALYs), clinical outcomes, and mortality. Most economic evaluations (75%) used QALY to measure health outcomes. Seventeen studies (50%) reported healthcare cost savings, although this cost decrease in comparison to usual care was only significant in nine studies. Regarding outcomes, twenty-two studies (65%) reported improved effectiveness in intervention groups. Of these, thirteen studies showed statistically significant improvement in outcomes compared to usual care.

### Results of main meta-analysis

**Fig. 2:**
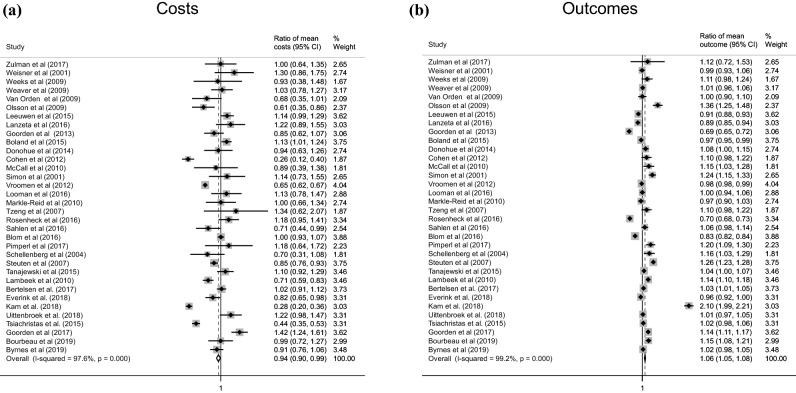
Mean differences in **a** costs and **b** outcomes between the intervention and control groups. A larger format is attached in Supplementary material Figures S2/3

Figure 2a shows the results of the meta-analysis on healthcare utilization costs. After meta-analysis of cost ratios with appropriate quality score-based weighting, the overall ratio of mean costs was 0.944 (95% CI 0.900–0.988) in favor of lower cost of the intervention group. The heterogeneity in ratio of mean costs across the studies was large (*I*^2^ = 97.6%). Although indicating a 5.6% decrease in cost as a result of care integration, the cost reduction was borderline statistically significant. Figure 2b illustrates the results of the meta-analysis of outcomes. The overall ratio of mean outcomes was 1.062 (95% CI 1.048–1.077), suggesting a 6.2% statistically significant improvement in outcomes associated with integrated care. However, the heterogeneity in the ratio of mean outcome across the studies was similarly large (*I*^2^ = 99.2%).

### Results of sub-group meta-analysis

Results from subgroup meta-analysis by study-duration, -design, -region and -type of integrated care intervention are shown in Table 1 (forest plots are displayed in Supplementary material Figures S2a–d and S3a–d). The subgroup analysis showed that integrated care was associated with lower cost and higher outcomes across all regions, although this association was not always statistically significant. Studies originating from Australia/Asia indicated the largest cost savings (ratio of means: 0.778; 95% CI 0.603–0.954) and health benefit (ratio of means: 1.426; 95% CI 1.376–1.477) followed by studies from Europe with ratio of mean costs of 0.951 (95% CI 0.905–0.997) and ratio of mean outcome of 1.025 (95% CI 1.015–1.035). Moreover, observational studies showed that integrated care was associated with lower cost (ratio of means: 0.781; 95% CI 0.687–0.874) and improvement in outcomes (ratio of means: 1.126; 95% CI 1.101–1.152). Neither the RCT nor Cluster RCT studies showed a significant association of integrated care with costs. However, RCTs showed a significant improvement in outcomes (ratio of means: 1.093; 95% CI 1.069–1.118) and Cluster RCT studies showed a significant worsening of outcomes (ratio of means: 0.922; 95% CI 0.908–0.937). Furthermore, studies lasting over 12 months showed a decrease in cost (ratio of means: 0.868; 95% CI 0.801 to 0.935) and improvement in outcomes (ratio of means: 1.148; 95% CI 1.112–1.184); whereas, these associations were not significant in studies with follow-up less than a year. Regarding the type of integrated care, the analysis reveals that disease management programs were associated with lower costs (ratio of means: 0.795; 95% CI 0.716–0.874) and improvement in outcomes (ratio of means: 1.114; 95% CI 1.093–1.135). The other types were associated only with either cost reduction (integrated care teams), cost increase (coordination between teams), or improved outcomes (integrated care management). Integrated care pathways showed no significant change in costs or effects.

**Table 1:** Pooled results of the meta-analysis of healthcare costs and hospitalization costs by subgroups

Characteristic	Subgroup	Study (*n*)	Mean weighted ratio of costs (mean intervention cost/mean control cost)	Mean weighted ratio of effects (mean intervention effect/mean control effect)
Region	North America	10	1.000 (95% CI 0.888–1.112)	1.029 (95% CI 0.981–1.077)
	Europe	20	0.951 (95% CI 0.905–0.997)	1.025 (95% CI 1.015–1.035)
	Australia/Asia	3	0.778 (95% CI 0.603–0.954)	1.426 (95% CI 1.376–1.477)
	Africa	1	0.695 (95% CI 0.309–1.082)	1.164 (95% CI 1.035–1.294)
Study design	RCT	17	0.993 (95% CI 0.930–1.057)	1.093 (95% CI 1.069–1.118)
	Cluster RCT	7	1.010 (95% CI 0.933–1.087)	0.922 (95% CI 0.908–0.937)
	Observational^a^	10	0.781 (95% CI 0.687–0.874)	1.126 (95% CI 1.101–1.152)
Study duration	> 12 months	12	0.868 (95% CI 0.801–0.935)	1.148 (95% CI 1.112–1.184)
	7–12 months	14	0.980 (95% CI 0.919–1.041)	0.998 (95% CI 0.985–1.011)
	≤ 6 months	7	1.002 (95% CI 0.877–1.126)	1.046 (95% CI 1.022–1.070)
	NA	1	0.929 (95% CI 0.383–1.475)	1.110 (95% CI 0.980–1.241)
Type of integrated care intervention	Integrated care team	9	0.901 (95% CI 0.824–0.979)	0.961 (95% CI 0.792–1.13)
	Coordination between services	7	1.160 (95% CI 1.033–1.286)	1.068 (95% CI 1.044–1.092)
	Integrated care management	10	0.914 (95% CI 0.817–1.012)	1.178 (95% CI 1.152–1.204)
	Integrated care pathways	4	0.922 (95% CI 0.845–1.000)	0.980 (95% CI 0.955–1.004)
	Disease management programs	4	0.795 (95% CI 0.716–0.874)	1.114 (95% CI 1.093–1.135)

^a^Observational studies include; pre-post cohort, cohort, and cross-sectional studies

## Discussion

### Findings and interpretation

This is the first meta-analysis of economic evaluations of integrated care across different clinical and care areas as well as types of integration. The results indicate that integrated care was associated with lower costs and improved outcomes compared with usual care, especially in studies with a follow-up period over a year. This may reflect the need for a sufficiently long follow-up period for effects to emerge, especially if there is a learning period following implementation [85]. In addition, studies with an extended follow-up period are more likely to capture long-term reductions in cost that may negate and surpass the initial investment in developing and implementing integrated care [86].

Moreover, our results raise questions about whether the long-term impact of integrated care has been captured in the economic evaluations. Decision analytic modeling that extrapolates costs and outcomes beyond study follow-up is recommended, especially when benefits of deliberating treatment plans for chronic diseases may be ongoing [28]. Nevertheless, it was performed in only one study [43]. Similarly, integrated care inherently intersects care boundaries and impacts a broad range of costs and outcomes within and beyond the healthcare system. However, only about a third of the economic evaluations included an (sensitivity) analysis taking the societal perspective, potentially missing more widespread cost savings of integrated care interventions related to costs in other sectors, informal care-giving, and productivity [87]. Taking the societal perspective in economic evaluations of integrated care is demanding and requires more complex and costly data collection. Although a health payer perspective remains the approach recommended by bodies such as the National Institute for Health and Care Excellence (NICE), this may jeopardize the quality of evidence about the cost-effectiveness of integrated care.

The pooled results of observational economic evaluations showed both significant reduction in costs and improvement in outcomes. However, this evidence was not found in studies with experimental designs. This contrast in the findings by study design highlights a well-documented trade-off between attributability and practicability [88]. In our review, several observational economic evaluations barely took any measures to mitigate for treatment contamination or selection bias, thereby jeopardizing causal inference [36, 52, 55, 64, 68, 69, 71, 74]. Although experimental designs are the gold standard for robust causal inference, their adoption in evaluating integrated care has been criticized due to their rigidness and low generalizability [89, 90].

Studies from Europe and Australia/Asia were significant in both costs and outcomes; whereas, studies from North America showed no significant effects. The reasons for this are unclear but could be owing to differences in healthcare systems [91] or the stage of implementation of integrated care interventions. North America is at a more advanced stage; it is possible, therefore, that the studies implemented are broader and involve larger populations, which may dilute the effects relative to smaller studies elsewhere where integrated care is still at a nascent stage [18].

Among types of intervention, disease management interventions alone showed significant decreases in costs and improvements in outcomes. This is similar to the findings of previous meta-analyses of disease management programs on single chronic conditions [20, 92, 93] and may mean that integrated care interventions are implemented more easily within single disease areas. Indeed, many initiatives around the world have started integrating services within single chronic conditions as a first step towards a wider integration [17]. Disease management programs have been long implemented in North America and Europe and certain levels of efficiency may have been achieved due to experience and productivity [94]. However, an important challenge remains, since disease management programs may not meet the needs of a patient with multiple health problems with complex needs [95].

### Quantity and quality of economic evaluations

Substantial investment into the implementation of integrated care is occurring on a global scale [96]. Despite this, only 34 economic evaluations of integrated care were identified in this review that had sufficient reported costs and outcomes to be included in the meta-analysis. Of these studies, only 19 (56%) had a quality score over 70%—a score above which is generally given to a study of “fair standard” [97]. The relatively low number of economic evaluations and their moderate methodological quality may stem from two reasons. First, economic evaluations are increasingly piggy backing effectiveness assessments of integrated care and are subject to insufficient communication between health economists and clinical/health service researchers [88]. Hence, regarding economic evaluations as an “afterthought” may be contributing to such a remarkably low number of suitable studies for meta-analysis. Second, integrated care interventions, like many complex interventions, are frequently not subject to extensive health technology assessment (HTA) as part of a reimbursement process at the national level. As a result, the cost-effectiveness of integrated care may receive less scrutiny than other health interventions (e.g., pharmaceuticals and clinical technologies) traditionally subject to HTA.

### Limitations

First, although the search strategy deployed aimed to include all studies broadly fitting the pre-set definition of integrated care, this review may have missed studies characterized by an alternative approach not covered by the search terms. Therefore, this review and meta-analysis may exclude some economic evaluations of interventions that could broadly fall under the integrate care umbrella term without explicitly fitting our working definition. However, it is expected that this may be the case for only a few studies as we have used broad concepts of integrated care in our search strategy. Second, despite the use of a binary system to review and assign quality scores to each economic evaluation based on the CHEERS-adapted checklist, there was opportunity for subjectivity which may have biased the scoring. Finally, due to the lack of reported standard error/ deviation of mean costs and outcomes, the heterogeneity across the studies reflected in the *I*^2^ statistic was based on the study quality rather than the precision of the mean [98]. Therefore, the statistical significance of the meta-analysis results should be interpreted with caution.

### Policy and research implications

Our findings support the reorientation of healthcare systems towards integration of care to help policy makers to meet increased demand for health and social care within tight budgets. However, with such sparse economic evaluations in integrated care, there is insufficient evidence about the factors that determine the cost-effectiveness of integrated care, such as models of care integration, implementation process, and target population. Efficiency of research is being streamlined in most other health innovations (e.g., pharmaceuticals and medical technologies) by including them in reimbursement processes with cost-effectiveness as an explicit criterion for market access [99]. Similar efforts should be made in assessing the cost-effectiveness of integrated care. Measures could be taken to standardize description of intervention and comparator, reporting of methods and results; apply appropriate follow-up periods and decision-analytic models; address bias; and deploy explicit decision criteria when value-for-money is uncertain. Such directed expansion of health economics towards the evaluation of integrated care is necessary to ensure decisions surrounding the implementation of integrated healthcare delivery are likely to benefit, rather than hinder, aims to meet increasing demands on tightening budgets.

## Electronic supplementary material

Below is the link to the electronic supplementary material.

Supplementary file1 (DOCX 374 kb)
