# Supplementary material for: Cost and effects of integrated care: a systematic literature review and meta-analysis
Source: Eur J Health Econ. 2020 Jul 6;21(8):1211–21. doi: 10.1007/s10198-020-01217-5 (PMC7561551; doi:10.1007/s10198-020-01217-5)
Supplement: Supplementary file 1 — Supplementary file1 (DOCX 374 kb) [file 10198_2020_1217_MOESM1_ESM.docx]

Supplementary material

Table of Contents

[Search terms 2](#_Toc35951214)

[Study characteristics 3](#_Toc35951215)

[Quality assessment 13](#_Toc35951216)

[Costs 14](#_Toc35951217)

[Outcomes 18](#_Toc35951218)

[Difference in outcomes and costs by subgroup 21](#_Toc35951219)

# Search terms

**Figure S1:**

**PUBMED/SCOPUS/EMBASE/MEDLINE/Ovid/CINAHL/NHS EED + Dare + Google searches:**

((‘integrated* care’) OR (‘integrated* delivery) OR (‘integrated* system’) OR (‘systems care’) OR ('transmural care') OR ( ('chains of care') OR ('collaborative* care') OR ('cooperative* care') OR ('coordinated* care') OR ('continuous* care') OR ('systems* care') OR ('intersectoral* care') OR ('interdisciplinary care’)l* care') OR ('cross sectoral* care') OR ('linked care') OR ('seamless care') OR ('shared care') OR ('transitional* care') (‘multidisciplinary care’) OR (‘linked* care’) OR (‘care linkages’) OR (‘care pathway’) OR (‘seamless care’) OR (‘shared care’) OR (’virtual integration*’) OR (‘horizontal integration*’) OR (‘functional integration*’) OR (‘normative integration*’) OR (‘collaborative* care’) OR (‘cooperative* care’) OR (‘information* continuity’) OR (‘interpersonal continuity’) OR (‘managed continuity’) OR (‘managed care’ OR (‘disease management’) OR (‘case management’)

AND (('cost utility*') OR ('cost effectiveness*') OR ('cost benefit') OR ('economic evaluation') OR ('cost impact') OR ('economic impact') OR ('cost savings*') OR ('cost consequence*') OR ('cost evaluation') OR ('economic appraisal') OR (‘cost comparison') OR (‘decision modelling’)

# Study characteristics

**Table S1: Study characteristics compiled from qualitative data abstraction**

|  | **Study characteristics** | | | | | | | | **Patient characteristics** | | | **Measures** |
| --- | --- | --- | --- | --- | --- | --- | --- | --- | --- | --- | --- | --- |
| Number | **Reference** | **Country** | **Study design** | **Type of economic evaluation** | **Perspective** | **Intervention Size** | **Control Size** | **Observation period** | **Target population** | **Setting** | **Study objective(s)** |  |
| 1 | Zulman et al (2017) | USA | RCT | Cost-consequence | Health care payer | 150 | 433 | 17 months | High healthcare users of (top 5%) | Primary care medical home | To evaluate the impact of augmenting the Veterans Affairs' medical home and multidisciplinary team with an intensive management program | Outcomes: 1) Patient satisfaction, 2) Patient activation measures; Cost: Inpatient and outpatient services |
| 2 | Weisner et al (2001) | USA | RCT | Cost-effectiveness | Health care payer | 285 | 307 | 6 months | Adults with alcohol and drug dependence | Primary care within substance abuse program | Examine differences in treatment outcomes and costs between integrated and independent models of medical and substance abuse care | Outcomes: 1) Alcohol and drug abstinence rate, 2) healthcare utilization (inpatient and outpatient) Cost: Inpatient, outpatient and treatment costs |
| 3 | Weeks et al (2009) | USA | Cross-sectional | Cost-comparison | Health care payer | 63,647 | 677,901 | NA | Individuals 65 years and older under Medicare | Multispecialty primary care group practice | Compare the costs and quality of care provided to Medicare beneficiaries by physicians who worked within large multispecialty physician group practices | Outcomes: 1) Outpatient clinical measures, 2) Ambulatory care-sensitive hospitalisations; Cost: Inpatient, long-term and home care |
| 4 | Weaver et al (2009) | USA | Cluster RCT | Cost-consequence | Health care payer | 232 | 199 | 12 months | Individuals with HIV, mental illness and substance abuse disorders | Outpatient multidisciplinary mental health, substance abuse and case management services | Evaluate the cost-effectiveness of integrating HIV primary care, mental health, and substance abuse services amongst triply diagnosed patients | Outcomes: 1) Quality of life, 2) mental health scores; Cost: Inpatient, outpatient, rehabilitation, home care, alternative, primary care, long-term care and out-of-pocket expenses |
| 5 | Van Orden et al (2009) | The Netherlands | Cluster RCT | Cost-consequence | Health care payer | 102 | 63 | 12 months | Adults with mental illness | Primary care and specialized mental health care | Compare the effect of introducing collaborative care on the attached–mental health professional model in a primary care setting | Outcomes: 1) Quality of life, 2) Satisfaction with care, 3) Mental health score; Costs: Treatment costs |
| 6 | Olsson et al (2009) | Sweden | Pre-post cohort | Cost-effectiveness | Health care payer | 56 | 56 | 18 months | Community dwelling older adults 65 years and older, with hip fracture | Multidisciplinary orthopaedic hospital ward | Compare costs and consequences of integrated care pathways for patients admitted with acute hip fractures | Outcomes: 1) Activities of daily living score; Cost: 1) intervention and operational costs, 3) implementation costs, 3) inpatient costs |
| 7 | Leeuwen et al (2015) | The Netherlands | Cluster RCT | Cost-utility | Societal perspective | 456 | 691 | 24 months | Community dwelling older adults with frailty | Multidisciplinary geriatric primary care team | Evaluate the cost-effectiveness of the Geriatric Care Model compared to usual primary care | Outcomes: 1) Quality adjusted life years, 2) Activities of daily living; Costs:1) inpatient, primary, outpatient, home and long-term care & medication costs, 2) informal care giver costs |
| 8 | Lanzeta et al (2016) | Spain | Cluster RCT | Cost-utility | Health care payer | 70 | 70 | 12 month | Individuals with multimorbidity | Primary and hospital based care | Examine the effectiveness of an integrated model for patients with multimorbidity, based on an assigned internist and a hospital liaison nurse | Outcomes: 1) Quality adjusted life years, 2) Health resource utilization; Costs: 1) acute, specialists, primary and home care, 2) Treatment costs |
| 9 | Goorden et al (2013) | The Netherlands | RCT | Cost-utility | Societal perspective | 65 | 61 | 12 months | Employees sick-listed due to major depressive disorder | Occupational health setting and consulting specialist care | Evaluate the cost-utility of a collaborative care intervention in sick-listed employees major depressive disorder | Outcomes: 1) Quality adjusted life years, 2) Health care utilization; Costs: 1) primary, specialist care and intervention costs, 2) Productivity loss |
| 10 | Boland et al (2015) | The Netherlands | Cluster RCT | Cost-utility | Societal perspective | 554 | 532 | 24 months | Patients with chronic obstructive pulmonary disease | Multidisciplinary primary care teams | Examine the cost-effectiveness of a disease management program for patients living with chronic obstructive pulmonary disease | Outcomes: 1) Quality adjusted life years, 2) symptom improvement; Costs: 1) acute, primary, rehabilitation and home care, 2) productivity loss and travel costs |
| 11 | Donohue et al (2014) | USA | RCT | Cost-utility | Health care payer | 150 | 152 | 12 months | Patients with depression following coronary artery bypass surgery | Primary care and specialized outpatient mental health care | Examine the impact of telephone-delivered collaborative care of treating post-CABG surgery depression compared to usual care | Outcomes: 1) Quality adjusted life years, 2) Depression free days; Costs: acute and outpatient costs |
| 12 | Cohen et al (2012) | Canada | Pre-post cohort | Cost-consequence | Societal perspective | 81 | Self comparator | 12 months | Children with medically complex chronic conditions | Outpatient clinics within community-based hospitals with paediatricians, linked with primary care | Evaluate the effectiveness of a community–based complex care clinic integrated with a tertiary care facility. | Outcomes: 1)Health related quality of life; 2) Perceptions of care; Cost; 1) inpatient, primary, outpatient and home care. 2) Out-of-pocket expenses for health and social care |
| 13 | Wise et al (2006) | USA | Cohort | Cost-comparison | Health care payer | 2010 | 30,360 | 12 months | Older adults , 65 years and older, with chronic conditions | Multidisciplinary primary care teams | Assess the impact of an integrated set of care coordination tools and chronic disease management interventions on utilization and cost | Cost: adjusted acute, primary care and drug costs |
| 14 | McCall et al (2010) | USA | Cohort | Cost-consequence | Health care payer | 2,619 | 2490 | 36 months | Older adults, aged 65 and older who are high cost medicare users | Multidisciplinary co-located primary care teams with linkages to home and long-term care | Evaluate whether the Massachusetts General Hospital (MGH) and its case management program can meet targeted cost-savings compared to control | Outcomes: 1) comorbidity score, 2) Care experience and satisfaction; 3) healthcare utilization; Cost: 1) covered inpatient, primary, outpatient and home care |
| 15 | Simon et al (2001) | USA | RCT | Cost-effectiveness | Health care payer | 110 | 109 | 6 months | Primary care patients with major depressive episode | Large primary care clinics part of a health cooperative | Evaluate the incremental cost-effectiveness of stepped collaborative care for patients with persistent depressive symptoms after usual primary care management. | Outcome: 1) Depression-free days; Costs: outpatient, primary, specialists and inpatient care |
| 16 | Hebert et al (2008) | Canada | Pre-post cohort (D-in-D) | Cost-consequence | Health care payer | 501 | 419 | 48 months | Older adults aged 65 living with frailty and disability | population level health and social care including: acute, home, long term, rehab and social services | Evaluate impacts of a progressively implemented PRISMA on the use of services and on costs in the experimental zone, compared with the comparison zone. | Outcomes: 1) functional and mental health scores; 2) care satisfaction; 3) care giver burden; Costs: 1) implementation and operation costs, 2) primary, specialist, acute and outpatient costs |
| 17 | Vroomen et al (2012) | The Netherlands | Cluster RCT | Cost-utility | Societal perspective | 201 | 136 | 6 months | Older adults living in residential homes | multidisciplinary residential home care linked with primary care | Evaluate the cost-effectiveness of a multidisciplinary integrated care in residential homes | Outcomes: 1) Quality adjusted life years, 2) Functional status, 3) Quality of care scores ; Costs: 1) acute, primary, outpatient/specialist care, 2) operational/implementation costs; 3) informal caregiver productivity loss |
| 18 | Salmon et al (2012) | USA | Pre-post cohort | Cost-comparison | Health care payer | 39,982 | Self comparator | 12 months | Patients enrolled collaborative accountable primary care organisations | primary care physician group practice | Examine the impact of accountable coordinated care initiative in three structurally and geographically diverse provider practices before and after implementation | Outcomes: 1) Outpatient/primary care clinical measures; Cost: 1) Inpatient and primary care, 2) intervention cost |
| 19 | Looman et al (2016) | The Netherlands | Pre-post cohort | Cost-utility | Societal perspective | 254 | 249 | 12 months | Community dwelling older adults with frailty | Multidisciplinary primary care team linked with nursing home and outpatient/specialist care | Examine the impact of integrated model for community-dwelling older adults with frailty | Outcome: 1) Quality adjusted life years; Cost: 1) inpatient, primary, home, outpatient and nursing home care, 2) intervention operational costs, 3) Informal care giver costs |
| 20 | Celano et al (2016) | USA | RCT | Cost-utility | Health care payer | 92 | 91 | 6 months | Patients hospitalized for cardiovascular illness with mental illness | Inpatient care followed by with telephone outpatient follow up, with primary care linkages | Examine the cost-effectiveness and differences in healthcare utilization and cost between collaborative depression and anxiety program with usual care | Outcomes: 1) Quality adjusted life years, 2) mental health status ; Costs: 1) acute, primary, outpatient/specialist care |
| 21 | Markle-Reid et al (2010) | Canada | RCT | Cost-consequence | Societal perspective | 55 | 54 | 6 months | Community dwelling older adults, 75 years and older, at risk for falls | Multidisciplinary home care linked with primary care and community services | Determine the effects and costs of a multifactorial, multidisciplinary team approach to falls prevention compared with usual home care services. | Outcomes: 1) Falls; 2) clinical outcomes (functional, mental and cognitive scores); 3) Quality adjusted life years; Cost: 1) Acute, home, primary and community care, 2) out-of-pocket indirect medical expenses |
| 22 | Pozzilli et al (2002) | Italy | RCT | Cost-consequence | Health care payer | 133 | 68 | 12 months | Patients diagnosed with multiple sclerosis | Multidisciplinary home care with specialists linkages | Compare the effectiveness and the costs of multidisciplinary home based care in multiple sclerosis with hospital care | Outcomes: 1) Quality of Life, 2) Health resource utilization; Costs: 1) inpatient, outpatient and home care, 2) intervention costs |
| 23 | Tzeng et al (2007) | China | RCT | Cost-consequence | Health care payer | 257 | 247 | 6 months | Individuals diagnosed with schizophrenia | Network of acute care, day hospital, rehabilitation, home care providers and local clinics | Compare the cost-effectiveness of an integrated model of schizophrenia treatment with those of the traditional treatment model provided by acute care | Outcomes: 1) Quality of Life, 2) Care giver burden, 3) Health service utilization, Costs: inpatient, outpatient, rehabilitation and home care |
| 24 | Bergmann et al (2017) | Malawi and Mozambique | Pre-post cohort (D-in-D) | Cost-effectiveness | Health care payer | Not reported | Not reported | 24 months | Children under 5 years with HIV who were underweight | Community based health workers and community clinics for HIV and acute under-nutrition | To estimate the impact and cost-effectiveness for integrated HIV and nutrition service delivery in sub-Saharan Africa. | Outcomes: 1) HIV infections averted, 2) Undernutrition cases cured, 3) Disability adjusted life years; Cost: 1) intervention costs, 3) operational/implementation costs, 2) life long HIV treatment cost |
| 25 | Koch et al (2017) | USA | Pre-post cohort (D-in-D) | Cost-comparison | Health care payer | 2.5 million per year | 2.5 million per year (their own control) | 15 months | Patients served by hospital and physician groups merged as part of horizontal integration of care | Hospital, primary and specialist care physicians | Assess how (financial) vertical integration affects volume and cost of services provided by acquired physicians and hospitals | Outcome: health care utilization; Cost: acute, primary and outpatient/specialist care |
| 26 | Rosenheck et al (2016) | USA | Cluster RCT | Cost-utility | Health care payer | 223 | 181 | 24 months | Individuals aged 15-40 in treatment for first episode of psychosis | Multidisciplinary community mental health treatment clinics | Compare the cost-effectiveness of a comprehensive, multidisciplinary, team-based treatment approach for first episode psychosis and usual Community Care in a cluster randomization trial | Outcomes: 1) Quality adjusted life years, 2) health service utilization; Costs: 1) inpatient, outpatient, residential and nursing home care and medication costs, 2) implementation and operational costs |
| 27 | Sahlen et al (2016) | Sweden | RCT | Cost-utility | Health care payer | 36 | 36 | 6 months | Patients diagnosed with congestive heart failure | Multidisciplinary palliative home care team linked with specialist | To assess the cost-effectiveness of person-centred integrated heart failure and palliative home care | Outcomes: 1) Quality adjusted life years; Costs: 1) acute, home, primary and specialist care, 2) intervention cost |
| 28 | Blom et al (2016) | Netherlands | RCT | Cost-consequence | Societal perspective | 3145 | 4133 | 12 months | Community-dwelling older adults, 75 and older living with complexity | General Practice with geriatric assessment training | Assess the effectiveness and cost- effectiveness of a monitoring system to detect the deterioration in somatic, functional, mental or social health, and followed by the execution of a care plan | Outcomes: 1) Quality of life, 2) activities of daily living, 3) satisfaction with care 4) Informal care giver time; Cost: 1) acute, primary, outpatient, nursing home and medication 2) intervention costs, 3) implementation costs, 4) informal care costs |
| 29 | Pimperl et al (2017) | Germany | Pre-post cohort | Cost-consequence | Health care payer | 5411 | 5411 | 48 months | Individuals enrolled with the accountable care organization insurance scheme | Cross-sectoral cooperation of physicians, hospitals, social care, nursing staff, therapists, and pharmacies | Identify an appropriate study design for evaluating population health outcomes of accountable care organization such as based on shared savings contract | Outcomes: 1) Survival, 2) comorbidity score; 3) Costs: outpatient physician and specialist care, hospital, rehabilitation, medication costs |
| 30 | Schellenberg et al (2004) | Tanzania | Cohort | Cost-consequence | Societal perspective | 100,000 | 100,000 | 24 months | Children with malaria, pneumonia, malnutrition and diarrhoea | Family and community primary care practices and hospitals | Assess the effectiveness of facility- based integrated management of childhood illness in rural Tanzania | Outcomes: 1) Child health outcomes, 2) Household health behaviour 3) Children’s mortality; Costs: 1) Drugs and vaccines, 2) Implementation costs & Operational costs, 3) intervention costs, 5) Out-of-pocket expenses, 7) Acute, primary and community care costs |
| 31 | Bird et al (2012) | Australia | Pre-post cohort | Cost-consequence | Health care payer | 223 | Self comparator | 36 months | Children with asthma that presented frequently at the emergency department | Acute, primary and other community-based care | Assess a model of care for paediatric asthma patients aimed to promote health and reduce their preventable acute care utilizations | Outcomes: 1) Activity limitation and emotional function, 2) acute care utilizations; Cost: 1) intervention costs; 2) acute care costs |
| 32 | Goltz et al (2013) | Germany | Cohort | Cost-consequence | Health care payer | 2455 | 2455 | 36 months | Patients with osteoporosis who experienced index fractures | Ambulatory care | Evaluate the outcomes of patients participating in a program of integrated care for osteoporosis in terms of medication supply, fracture incidence and expenses | Outcomes: 1) Fracture Incidence, 2) Occurrence of pain; Costs: 1) Acute care costs, 2) treatment costs, 3) medication costs |
| 33 | Steuten et al (2007) | Netherlands | pre-post cohort | Cost-utility | Societal perspective | 2455 | 2455 | 60 months | Patients 18 years and older with GP diagnosis of asthma | Primary care collaborating with specialists | Assess long-term cost-utility of a disease management program for adults with asthma was assessed compared to usual care | Outcomes: 1) Quality adjusted life years, 2) asthma related exacerbations/control Costs: 1) acute, primary and outpatient/specialist care costs, 2) medication costs 3) treatment costs, 4) implementation and operational costs, 5) patient productivity loss |
| 34 | Wiley-Exley et al (2009) | USA | Cluster RCT | Cost-utility | Societal perspective | 1257 | 1948 | 6 months | Older adults, 65 years and older with major depressive disorder in primary care | Multidisciplinary specialist team co-located in primary care | Compare the cost-effectiveness of integrated care in primary care to enhanced specialty referral for older adults with behavioural health disorders | Outcomes: 1) Depression free days, 2) Quality Adjusted Life Years; Costs: 1) inpatient, emergency room use, nursing home, rehabilitation care. 2) medication costs, 3) caregiver and patient indirect costs (transportation, productivity loss) |
| 35 | Karow et al (2012) | Germany | Cohort | Cost-utility | Health care payer | 64 | 56 | 12 months | Adults patients diagnosed with first or multiple-episode schizophrenia | Inpatient, outpatient/specialists and occupational therapy care | To compare the cost effectiveness of therapeutic assertive community treatment with standard care in schizophrenia. | Outcomes: 1) Quality adjusted life years, Costs: 1) inpatient care, day-clinic care, outpatient and specialist care costs, 2) medication costs |
| 36 | Renaud et al (2009) | Burundi | Cohort | Cost-effectiveness | Health care payer | 149 | Self comparator | 60 months | People living with HIV who initiated antiretroviral treatment | Primary care based on non-for profit organization delivering care for individuals living with HIV | Calculate the incremental cost effectiveness of an integrated care package for people living with HIV/AIDS in a not-for-profit primary health care centre. | Outcomes: 1) Disability adjusted life years Cost: 1) outpatient, acute and home care costs 2) medication costs 3) intervention costs, 4) food support costs |
| 37 | Tanajewski et al (2015) | United Kingdom | RCT | Cost-utility | Health care payer | 205 | 212 | 3 months | Older people at risk of adverse outcomes after acute care discharge | Multidisciplinary acute care team and links to primary care | To examine the cost-effectiveness of a specialist geriatric medical intervention for frail older people in the 90 days following discharge from an acute medical unit | Outcomes:  1) Quality adjusted life years; Costs: 1) acute, primary and specialist care; 2) intervention costs |
| 38 | Lambeek et al (2010) | Netherlands | RCT | Cost-utility | Health care payer | 66 | 68 | 12 months | Individuals visiting outpatient clinic due to low back pain | Occupational health setting linked with multidisciplinary outpatient team | To evaluate the cost effectiveness of an integrated occupational health programme for sick listed patients with chronic low back pain. | Outcomes: 1) Duration until sustainable return to work; 2) Quality adjusted life years; Costs: 1) primary and secondary care, home care, and drugs.  2) Out of pocket expenses for additional and informal care; 3) patient productivity loss |
| 39 | Bertelsen et al. (2017) | Denmark | RCT | Cost-utility | Societal perspective | 106 | 106 | 12 months | Adult patients admitted to the hospital with acute coronary syndrome | Shared care between primary care and outpatient public health centres, with multidisciplinary teams | To assess the cost-utility of shared care cardiac rehabilitation versus hospital-only cardiac rehabilitation from a societal perspective | Outcomes: 1) Quality-adjusted life years; Costs: 1) intervention cost/ formal and informal staff time; 2) primary and secondary care; 3) productivity losses |
| 40 | Camacho et al. (2018) | United Kingdom | Cluster RCT | Cost-utility | Health care payer | 191 | 196 | 24 months | Patients with depressive symptoms and a record of diabetes and/or coronary heart disease | General practices with case managers co-located with multidisciplinary team | To assess the cost-effectiveness of collaborative care for people with depression in the context of multimorbidity | Outcomes: 1) depression severity; 2) Quality adjusted life years; 3) health care utilization Costs: 1) inpatient; 2) outpatient; 3) emergency; 4) primary/community care; 5) intervention costs; 6) Implementation and training costs |
| 41 | Everink et al. (2018) | The Netherlands | Cohort | Cost-utility | Societal perspective | 113 | 49 | 9 months | Community-dwelling older patients who were admitted to a geriatric rehabilitation facility | Coordination between the hospital, the geriatric rehabilitation and primary care and home care | To determine the cost-effectiveness of receiving usual care to receiving care in the integrated care pathway | Outcomes: 1) dependence in activities of daily living; 2) Quality adjusted life years; Cost: 1) intervention costs; 2) implementation costs; 3) primary, home care, long-term care, acute care and allied professionals; 4) patient out-of pocket expenses; 5) informal caregiving |
| 42 | Kam et al. (2018) | China | RCT | Cost-utility | Health care payer | 43 | 41 | 24 months | End-stage heart failure patients referred to in-hospital palliative care services | Transitional care between hospital to home care delivered by case manager and multidisciplinary home care team | To evaluate the cost-effectiveness of a transitional home-based palliative care program | Outcomes: 1) Quality adjusted life years; Costs: 1) acute, home and emergency care; 2) intervention cost; 3) training cost |
| 43 | Uittenbroek et al. (2018) | The Netherlands | RCT | Cost-utility | Societal perspective | 747 | 709 | 12 months | Older adults, aged 75 and over with primary care providers | General practitioner-led elderly multidisciplinary care team in primary care with case manager | To assess the cost-effectiveness of integrated geriatric care team in primary care | Outcomes: 1) Quality adjusted life years; 2) number of days older adult was able to age in place (i.e. no nursing home stays); Costs: 1) primary, acute, medication and paramedical care; 3) social and home care; 4) informal caregiving |
| 44 | Tsiachristas et al. (2015) | The Netherlands | Cohort | Cost-utility | Societal perspective | 1034 | 1034 | 24 months | patients diagnosed with or at risk of cardiovascular disease and chronic obstructive pulmonary disorder | Disease management programs implemented through collaborations between general practices and hospitals, primary care practices (including physiotherapists and dieticians), or primary and community settings) | To evaluate the cost-effectiveness of disease management programs for patients diagnosed with or at risk of cardiovascular disease and chronic obstructive pulmonary disorder | Outcomes: 1) Quality adjusted life years; 2) level of physical activity; 3) proportion of smokers; Costs: 1) health care utilization costs; 2) travel costs; 3) productivity loss; 4) development costs; 5) implementation costs |
| 45 | Goorden et al (2017) | The Netherlands | multicenter RCT | Cost-utility | Health care and societal perspective | 42 | 39 | 12 months | patients with a chronic physical condition and major depressive disorder (MDD) | outpatient general hospital setting | to assess the costutility of CC for the treatment of comorbid MDD in chronic medically ill patients in the outpatient general hospital setting from a societal perspective, taking all relevant costs and effects into account | Outcomes: 1) TiC-P, 2) short-form Health-Related Quality of Life questionnaire, 3) EuroQol EQ-5D 3 level version, measuring the use of health care, informal care, and household work |
| 46 | Bourbeau et al (2019) | EU | multicenter RCT | Cost-utility | Health care payer | 157 | 162 | 12 months | Patients with severe COPD | Home-based disease management (DM) program compared with usual management (UM) in four European countries | to perform a cost-effectiveness evaluation, based on COMET, comparing DM with the current UM | Outcomes: 1) survival, 2) quality of life (QoL), and 3) direct medical and nonmedical costs |
| 47 | Byrnes et al (2019) | Australia | multicenter RCT | Cost-utility | Health care payer | 168 | 167 | 24 months | Patients with a diagnosis of paroxysmal persistent and permanent forms of non-valvular Atrial Fibrilation | Participants were randomized to receive either standard post-discharge management or an AF-specific nurse-led, home-based management strategy involving comprehensive health assessment, risk delineation, individualized care, and annual clinic visits | to ascertain the cost-effectiveness of the SAFETY Atrial Fibrillation management strategy | Outcomes: 1) all-cause death, 2) unplanned re-hospitalization due to any reason,3) Quality of life, 4) health service use, including: hospitalization episodes, outpatient specialist and general practitioner (primary care physician) attendances, , and prescription medications reimbursed by the government |

# Quality assessment

**Table S2: Quality Assessment**

# Costs

**Table S3: Costs**

| **Number** | **Study** | **Year used for cost** | **Currency** | **Costs included in total cost** | **Mean total cost per patient (intervention)** | **standard error of mean total cost per patient (intervention)** | **Mean total cost per patient (comparator)** | **standard error of mean total cost per patient (comparator)** | **Incremental cost** | **ICER** |
| --- | --- | --- | --- | --- | --- | --- | --- | --- | --- | --- |
| 1 | Zulman et al (2017) | 2013 | $ | total cost of VA care (including inpatient, outpatient, and fee-basis care) and cost of specific inpatient services/ outpatient services and program costs incurred by the facility | monthly cost declined ﻿21.0%, from baseline measurement $6139 to $4850 | NA | ﻿monthly cost declined 20.7%, from baseline measurement $5821 to $4618 | NA | difference in differences = –$101 (st error: $623) | NA |
| 2 | Weisner et al (2001) | - | $ | unit cost of services, and outside appointments (weighted) | $428.87 | ﻿95% CI: 397.63-460.11 | $382.81 | ﻿95% CI: 352.59-413.03 | -100.06 | ﻿$1581 per additional person abstinent in integrated services relative to independent services |
| 3 | Weeks et al (2009) | 2005 | $ | ﻿mean standardized physician spending per Medicare patient; mean standardized inpatient spending per patient (includes acute hospitalizations, long-term care facilities, and skilled nursing facilities); mean standardized home health care costs per patient; and mean total standardized Centers for Medicare and Medicaid Services (CMS) program payments per patient. | Crude measures: $7,053 | NA | Crude measures: ﻿$7,593 | NA | Crude measures: ﻿−$540 (95% CI: ﻿−$627﻿ to ﻿−$452) Adjusted measures: ﻿−$272 (95% CI: −$355 to ﻿−$189) | NA |
| 4 | Weaver et al (2009) | 2002 | $ | inpatient/outpatient services and medication | 3235 (baseline) to 3052 (12 month) and hence -183 difference; 0.94 ratio (baseline 3 month average over 12 month average cost) | Test change in intervention group over time: p=0.41 | 3556 (baseline) to 3271 (12 month) and hence -285 difference; 0.92 ratio (baseline 3 month average over 12 month average cost) | ﻿Test change in control group over time: p=0.81 | Difference in differences: 102 ﻿ | NA |
| 5 | Van Orden et al (2009) | NA | € | GP and mental health services | ﻿€1,199±1,621 | NA | ﻿€1,762±1,683 | NA | $563 | NA |
| 6 | Olsson et al (2009) | 2004 | € | hotel and treatment cost | ﻿9685 (9629-->5% trimmed cost) | ﻿2219= st dev | ﻿15,984 (15,645-->5% trimmed cost) | ﻿7959= st dev | $6299 (﻿4255- 8828 = 95% CI of the difference) | ﻿€14,840 per rehabilitated patient (cost effectiveness ratio of intervention) and ﻿€31,908 per rehabilitated patient (cost effectiveness ratio of control) |
| 7 | Leeuwen et al (2015) | 2011 | $ | primary and community care, societal cost, secondary care | ﻿11,659 | 329= st error | ﻿10,207 | 408 = st error | Adjusted mean difference: ﻿$356 (95% CI = -$488–1,134) Crude mean difference: $1452 | 842 |
| 8 | Lanzeta et al (2016) | NA | € | hospitalisations, primary care and specialised care | ﻿6,066.4 | ﻿5,514.8= st dev | ﻿4,973.3 | ﻿5,486.4= st dev |  |  |
| 9 | Goorden et al (2013) | 2009 | € | ﻿measuring the number of contacts with health care services during the last 3 months, which can then be multiplied by the reference unit prices of 2009 of these services and productivity losses | ﻿3,874 | (﻿95 % CI €2,778– €5,718) | ﻿4,583 | ﻿95 % CI €3,108–€6,794 | 709 | ﻿€14,589 per QALY |
| 10 | Boland et al (2015) | 2013 | € | ﻿medica- tion prescriptions, contact with care providers, home care, hospital admissions, emergency department visits, pulmonary rehabilitation, societal costs and travel costs | 5119 (healthcare perspective) and 5750 (societal perspective) | NA | 4535 (healthcare perspective) and 5105 (societal perspective) | NA | 584 (difference from healthcare perspective with 95% CI: 86 to 1046) and 645 (difference from societal perspectivewith 95% CI: 28 to 1190) | –15720 per QALY (healthcare perspective) and –17358 per QALY (societal perspective) |
| 11 | Donohue et al (2014) | NA | $ | healthcare payer costs (inpateint/outpatient costs in the context of relative reimbursement data of each insurance company) | ﻿16,552 | ﻿18,050= st dev | ﻿17,522 | ﻿21,072= st dev | –971 with p=0.69 | −$21,591 (95% CI:−$26,064 to −$17,118) |
| 12 | Cohen et al (2012) | 2009 | $ | ﻿Costing estimates incorporated both third-party payer (Ontario’s Ministry of Health and Long-Term Care) and parental perspectives (out-of-pocket costs). | 369 | 708 | ﻿1439 | 3511= st dev | 1070 | NA |
| 13 | Wise et al (2006) | NA | $ | ﻿true relative costs between the MMC and the comparison population resulting from differences in the healthcare service utilization | 188 | NA | 436 | NA | 248 | NA |
| 14 | McCall et al (2010) | NA | $ | ﻿Medicare program Part A and B payments;﻿claims for utilization of beneficiaries when they are eligible for the demonstration; | 1500 at baseline year then 2022 average per month over 36 months | 41.8= st error at baseline; 47.9= st error at 36 months | 1563 at baseline year then 2373 average per month over 36 months | 46.1= st error at baseline; 54.2= st error at 36 months | 63 at baseline and 351 at 36 months | NA |
| 15 | Simon et al (2001) | NA | $ | outpatient and inpatient services including prescriptions | ﻿2,406 | ﻿95% CI: 1,769–3,218 | ﻿2,110 | 95% CI: 1,503–3,234 | ﻿$357 | 35.05 per depression free day (95%CI: ﻿–51.73 to 387.54) |
| 16 | Hebert et al (2008) | NO COSTS REPORTED | NA | NA | NA | NA | NA | NA | NA | NA |
| 17 | Vroomen et al (2015) | 2010 | € | community based health services | 69,435 total cost | NA | 107627 total cost | NA | 38192 (CI: –85,606 to 9,222) | 9581433 per QALY |
| 18 | Salmon et al (2012) | 2009 | $ | outpatient and inpatient services including prescriptions | NA | NA | NA | NA | only gave very limited graphicla data on the difference in cost performance of the control and integrated groups | NA |
| 19 | Looman et al (2016) | 2011 | € | inpatient/outpatient services and medication and informal care costs | ﻿17 089 | st dev=﻿21 468﻿ | ﻿15 189 | st dev=﻿21 709 | 1970 | ﻿412 450 per QALY |
| 20 | Celano et al (2016) | NA | $ | ﻿Hospitalizations,Emergency department visits, Psychiatric visits, Therapy visits,Primary care, physician visits, Cardiologist visits, Psychiatric medications | ﻿24,596.43 | ﻿z=1.14; P= 0.25 | ﻿21,912.85 | ﻿z=1.14; P= 0.25 | 2683.85 | ﻿$3337.06 per QALY, $13.36 per DFD, and $13.74 per AFD. |
| 21 | Markle-Reid et al (2010) | 2006 | $ | inpatient/outpatient services and medication and pensions/ benefits | NA | NA | NA | NA | ﻿The mean six-month costs of use of all types of health services decreased overall by 78.3 per cent (from $22,956 at baseline to $4,973 at six months). The change in total per-person direct costs of use of health services did not differ between the two groups ( p = 0.41) | NA |
| 22 | Pozzilli et al (2002) | 1999 | € | ﻿Inpatient care, outpatient and home care, non-medical care, home care programme (intervention only) | 1443 | NA | 2265 | NA | -822 | NA |
| 23 | Tzeng et al (2007) | 2002? | $ | inpatient/outpatient services and medication and home care | 2737 | NA | 2041 | NA | 696 |  |
| 24 | Bergmann et al (2017) | 2012 | $ |  |  |  |  |  |  |  |
| 25 | Koch et al (2017) | NA | $ | Hospital running costs | 136347 | st dev: 150,268 | 114689 | st dev: 119,465 | 21958 |  |
| 26 | Rosenheck et al (2016) |  | $ | ﻿Services and medication using generic APS costs and training costs | 7088 | 1127 | 6019 | 1260 | 1069 | ﻿$14 696/QLS-SD |
| 27 | Sahlen et al (2016) | 2012 | € | inpatient/outpatient services and emergency transport | 4078 | NA | 5727 | NA | –1649 |  |
| 28 | Blom et al (2016) | NA | € | Screening, carrying out of care-plan, planning of care plan, training | 236 | NA | 236 | NA | 0 | NA |
| 29 | Pimperl et al (2017) |  | € | Outpatient physician and specialist care, hospital, medical inpatient rehabilitation, medicatio | 1961 | st dev: 5145 | 1661 | st dev: 3465 |  |  |
| 30 | Schellenberg et al (2004) |  | Tsh | National, district, household, hoptial and primary facility care cost | 8695 | NA | 12503 | NA | 3808 |  |
| 31 | Bird et al (2012) |  | $ | NA | NA | NA | NA | NA | NA |  |
| 32 | Goltz et al (2013) |  |  |  |  |  |  |  |  |  |
| 33 | Steuten et al (2007) |  | € |  | 3242 | NA | 3833 | NA | NA | ﻿approxi- mately €1,000 for an additional QALY |
| 34 | Wiley-Exley et al (2009) |  | $ | Outpatient, inpatient, home care and transport services | ﻿$3,657 | NA | ﻿$3,673 | NA | –669 | ICER illustrated on plane |
| 35 | Karow et al (2012) |  |  |  |  |  |  |  |  |  |
| 36 | Renaud et al (2009) |  |  |  |  |  |  |  |  |  |
| 37 | Tanajewski et al (2015) |  | $ | Initial Assessment Home visits Phone calls Clinic visitse | 4475 | NA | 4057 | NA | 418 | ﻿£147 087/QALY |
| 38 | Lambeek et al (2010) |  | $ | Primary, secondary, and home care, drugs and direct non-healthcare costs | 13165 | 13600 | 18475 | 13616 | 5310 | –$61,000/QALY |
| 39 | Bertelsen et al. (2017) | 2013 | DKK | Opportunity cost, CR provision cost, healthcare use in primary care and in hospitals, patient time, travvel costs, and productivity losses due to inability to participate in the labour force. | 165475 | NA | 163000 | NA | –2475 (95% CI of difference: −38 101 to 43 052) | NA |
| 40 | Camacho et al. (2018) |  |  |  |  |  |  |  |  |  |
| 41 | Everink et al. (2018) | 2014 | € | Costs from societal perspective: Health care use, assistive devices, environmental adaptations and travel expenses | 50791.38 | 2473.7 | 62169.59 | 4807.91 | –11605 | –11186 per KATZ-15 and –2,304,876 per QALY |
| 42 | Kam et al. (2018) | 2015 | HK$ | Outpatient,inpatient and training costs | 10123 | NA | 36206 | NA | –26083 | NA |
| 43 | Uittenbroek et al. (2018) | 2012 | € | cost to health insurance, medical care, social care | 13073 | 18104 | 10677 | 14476 | –2396 | NA |
| 44 | Tsiachristas et al. (2015) | 2012 | € | Healthcare perpective: the costs of health care utilization;he development costs of the DMPs; and 5) the implementation costs of the DMPs. Societal cost: the costs borne by patients for traveling to receive care; the costs of productivity loss due to absence from paid work. | 2741 societal perspective and 2509 healthcare perspective | 95% CI: 1,867 to 3,995 from societal perspective and 95% CI: 1,563 to 4,085 from healthcare perpective | 8,007 societal perspective and 5,730 healthcare perspective | 95% CI: 2,715 to 19,3856 from societal perspective and 95% CI: 1,946 to 12,156 from healthcare perpective | –5,266 societal perspective and –3,221 healthcare perspective | |
| 45 | Goorden et al (2017) | 2016 | € | direct costs (clinical consultation costs, outpatient costsl) indirect costs (informal care and household work) societal perspective | 6718 societal perspective and 6522 healthcare perspective | 95% CI: 3,541 to 10,680 from societal perspective and 95% CI: 3,239 to 10,760 from healthcare perpective | 5038 societal perspective and 4582 healthcare perspective | 95% CI: 3,159 to 7,346 from societal perspective and 95% CI: 2,782 to 6,740 from healthcare perpective | 1,680 societal perspective and 1,939 healthcare perspective | 24,690/QALY societal perspective and 28,366/QALY from healthcare perspective |
| 46 | Bourbeau et al (2019) | 2015 | € | Direct medical and nonmedical costs, including those associated with unscheduled hospitalizations, outpatient visits, coaching by case managers, and management of the alerts and alarms by the case managers and investigators, were accounted for each patient in the ITT population. Technical costs relating to the set-up of the DM intervention included both fixed (not related to the number of patients in the group) and variable costs (related to the number of patients in the group). | 7019 |  | 7056 |  | −37 | dominant |
| 47 | Byrnes et al (2019) | 2015 | A$ | Hospitalization episodes, outpatient specialist and general practitioner (primary care physician) attendances, and prescription medications reimbursed by the government. The costs per person of the intervention and standard management groups were estimated based on the number of clinic visits, home visits, and telephone contacts. | 43720 |  | 48095 |  | -4375 | Dominant |

# Outcomes

**Table S4: Outcomes**

| **NUMBER** | **Study** | **Measurement of quality of life** | **Mean quality of life(intervention)** | **Standard error of mean quality of life (intervention)** | **Mean quality of life (comparator)** | **Standard error of mean quality of life (comparator)6** | **Difference in quality of life** | **Baseline adjustment** |
| --- | --- | --- | --- | --- | --- | --- | --- | --- |
| 1 | Zulman et al (2017) | Mortality rates during the programme | 12.1 | NA | 13.6 | NA | ﻿1.4% [95% CI: −5.7% to 7.3%] |  |
| 2 | Weisner et al (2001) | SF-12 | 49.6 | st dev: 10.5 | 49.9 | st dev: 9.9 | -0.3 |  |
| 3 | Weeks et al (2009) | Completion rate of all three diabetes tests | 63.4 | NA | 57.1 | NA | relative risk ratio: 1.12 (CI: 1.10-1.16) |  |
| 4 | Weaver et al (2009) | SF-6D | 0.611 | NA | 0.606 | NA | 0.005 (CI: –0.011 to 0.021 and P-value 0.539) | |
| 5 | Van Orden et al (2009) | WHOQOL-BREF | 3.0 at baseline and 3.3 at 12 months | st dev at baseline: 0.8 ; st dev at 12 months: 0.9 | 3.0 at baseline and 3.3 at 12 months | st dev at baseline: 1.0 ; st dev at 12 months: 0.7 | No difference |  |
| 6 | Olsson et al (2009) | %patients rehabilitated | 75 | NA | 55 | NA | 0.2 |  |
| 7 | Leeuwen et al (2015) | QALY | 0.29 | 0.003 | 0.32 | 0.004 | 0.003 (95% CI: ﻿–0.006 to 0.012) |  |
| 8 | Lanzeta et al (2016) | QALY | ﻿0.4682 | st dev= ﻿0.4003 | ﻿0.5235 | st dev=﻿0.3886 | 0.0117 |  |
| 9 | Goorden et al (2013) | Improvement in QALY | 0.11 | 95% CI : ﻿﻿0.07–0.14 | 0.16 | 95% CI : ﻿0.11 to 0.19 | ﻿-0.05 QALY |  |
| 10 | Boland et al (2015) | number of QALYs over the two year period | 1.4 | NA | 1.44 | NA | –0.04 (95% CI: –0.07 to –0.01) |  |
| 11 | Donohue et al (2014) | QALY | ﻿0.70 | p=0﻿.004 | ﻿0.65 | p=0.004 | 0.05 |  |
| 12 | Cohen et al (2012) | Peds QL | ﻿47.9 | 20.3= st dev | ﻿43.6 | 14.3 = st dev | 4.3 |  |
| 13 | Wise et al (2006) | HEDIS | see image 6- unclear |  |  |  |  |  |
| 14 | McCall et al (2010) | mortality rates during the programme | original population: 27.09% refresh population: 15.6% | NA | original population: 29.5 refresh population : 19.6 | NA | original population difference: –1.63 (p value 0.19) refresh population difference: –3.97 (p value 0.04) | |
| 15 | Simon et al (2001) | mean number of depression free days | 87.7 | ﻿95% CI= 76.6–96.7 | 70.9 | ﻿(95% CI=60.8–81.3 | 16.7 |  |
| 16 | Hebert et al (2008) | SMAF score /87 | 18.1 at baseline to 26.6 at four years | 95% CI: 17.0–19.1 at baseline when n=419 and 95%CI: 25.5–27.8 at four years when n=434 | 17.0 at baseline to 25.9 at four years | 95% CI: 16.0-18.0 at baseline when n=501 and 95%CI: 24.7–27.1 at four years when n=395 | Comparison of the evolution over time between groups standard vs collaborative =0.684 | |
| 17 | Vroomen et al (2015) | QALY | 1.25 | 0.04 | 1.27 | 0.04 | –0.02 |  |
| 18 | Salmon et al (2012) | ﻿Percentage-point difference between initiative practice and its comparison group, compliance across all standards 2010 | NA | NA | NA | NA | 0.7, 2.6, 4.7 |  |
| 19 | Looman et al (2016) | Effects on EQ-5D over the 12 months | 0 | 0.19 | –0.01 | 0.17 | 0.01 |  |
| 20 | Celano et al (2016) | QALY | NA | NA | NA | NA | ﻿CC group experienced 0.053 more QALYs (t =2.49; P =0.01) than did subjects in the EUC group over the 24-week follow-up period | |
| 21 | Markle-Reid et al (2010) | ﻿Number of Falls in Past Six Months | baseline=﻿1.76 ; sixth month follow up =﻿1.45; difference = 0.31 | st dev for baseline= 2.72; st dev for six months= 2.73; st dev for difference= 2.55 | baseline=﻿1.67 ; sixth month follow up =﻿1.33; difference = 0.35 | st dev for baseline= 3.74; st dev for six months= 2.23; st dev for difference= 3.34 | difference in mean change scores –0.04 (95% CI= ﻿−1.27, 1.18) | √ |
| 22 | Pozzilli et al (2002) | DIFFERENCE in SF-36 Physical Composite Score | NA | NA | NA | NA | difference in mean change 1.19 | √ |
| 23 | Tzeng et al (2007) | Quality of life questionnaire based upon WHOQOLBRIEF | baseline=﻿81.32 ; follow up =﻿81.75; difference = 0.43 | NA | baseline=﻿78.93 ; follow up =﻿76.56; difference = –2.37 | NA | difference in mean change: 2.8 | √ |
| 24 | Bergmann et al (2017) | DALY |  |  |  |  |  |  |
| 25 | Koch et al (2017) | NA | NA | NA | NA | NA | NA |  |
| 26 | Rosenheck et al (2016) | ﻿Difference in QLS-SD from baseline to follow up | 0.31 | NA | 0.24 | NA | difference in mean change: 0.07 | √ |
| 27 | Sahlen et al (2016) | EQ-5D to measure QALY | baseline=﻿0.596 ; follow up =﻿ 0.602; difference = 0.006 | NA | baseline=﻿0.538 ; follow up =﻿ 0.514; difference = –0.024 | NA | difference in mean change: 0.03 | √ |
| 28 | Blom et al (2016) | DIFFERENCE in GARS total score | 2.9 | NA | 3.5 | NA | difference in mean change: –0.6 | √ |
| 29 | Pimperl et al (2017) | % mortality of patients across 4 years | 4.1 | NA | 4.9 | NA | 0.8 |  |
| 30 | Schellenberg et al (2004) | Mortality rates during the programme | baseline=﻿ 27.2 ; follow up =﻿ 24.4; difference = –2.8 | NA | baseline=﻿27.0 ; follow up =﻿ 28.2; difference = 1.2 | NA | difference in mean change: 4.0 | √ |
| 31 | Bird et al (2012) | PACQOL | 25.4 | st dev: 5.0 | 19.8 | st dev: 7.1 | 5.6 |  |
| 32 | Goltz et al (2013) | Median uptake of analgesics | 30 | NA | 35.5 | NA | NA |  |
| 33 | Steuten et al (2007) | QALY | 3.4 | At baseline: 0.31 | 2.7 | NA | 0.7 |  |
| 34 | Wiley-Exley et al (2009) | Change in QALY-DFD |  |  | 2.9 |  |  |  |
| 35 | Karow et al (2012) |  |  |  |  |  |  |  |
| 36 | Renaud et al (2009) |  |  |  |  |  |  |  |
| 37 | Tanajewski et al (2015) | QALYs gained | 0.107 | NA | 0.103 | NA | difference in mean change: 0.004 | √ |
| 38 | Lambeek et al (2010) | Change in QALY | 0.74 | st dev: 0.19 | 0.65 | st dev: 0.21 | Difference in mean change:0.09 | √ |
| 39 | Bertelsen et al. (2017) | QALY | 0.849 | NA | 0.826 | NA | 0.023 |  |
| 40 | Camacho et al. (2018) |  |  |  |  |  |  |  |
| 41 | Everink et al. (2018) | EQ-5D-3L Dutch tariff mean score (range -0.329–1 ) | 0.51 | 0.3 | 0.53 | 0.28 | 0.01 |  |
| 42 | Kam et al. (2018) | QALYs gained | 0.0147 | NA | 0.007 | NA | difference in mean change: 0.77 | √ |
| 43 | Uittenbroek et al. (2018) | QALY | 0.77 | 0.2 | 0.76 | 0.22 | 0.01 |  |
| 44 | Tsiachristas et al. (2015) | QALY | 0.777 | 95% CI: 0.749–0.806 | 0.76 | 95% CI: 0.709–0.804 | 0.017 |  |
| 45 | Goorden et al (2017) | QALY | at baseline: 0.43 gained: 0.07 | at baseline: 0.31 | at baseline: 0.45 gained: 0.01 | at baseline: 0.28 | difference in QALY gained: 0.06 |  |
| 46 | Bourbeau et al (2019) | QALY | 0.772 |  | 0.673 |  | 0.099 |  |
| 47 | Byrnes et al (2019) | QALY | 1.22 |  | 1.2 |  | 0.02 |  |

# Difference in outcomes and costs by subgroup

**Figure S2: Ratio of mean costs by (a) region (b) study design (c) study duration and (d) type of integrated care intervention**

2(a)

**
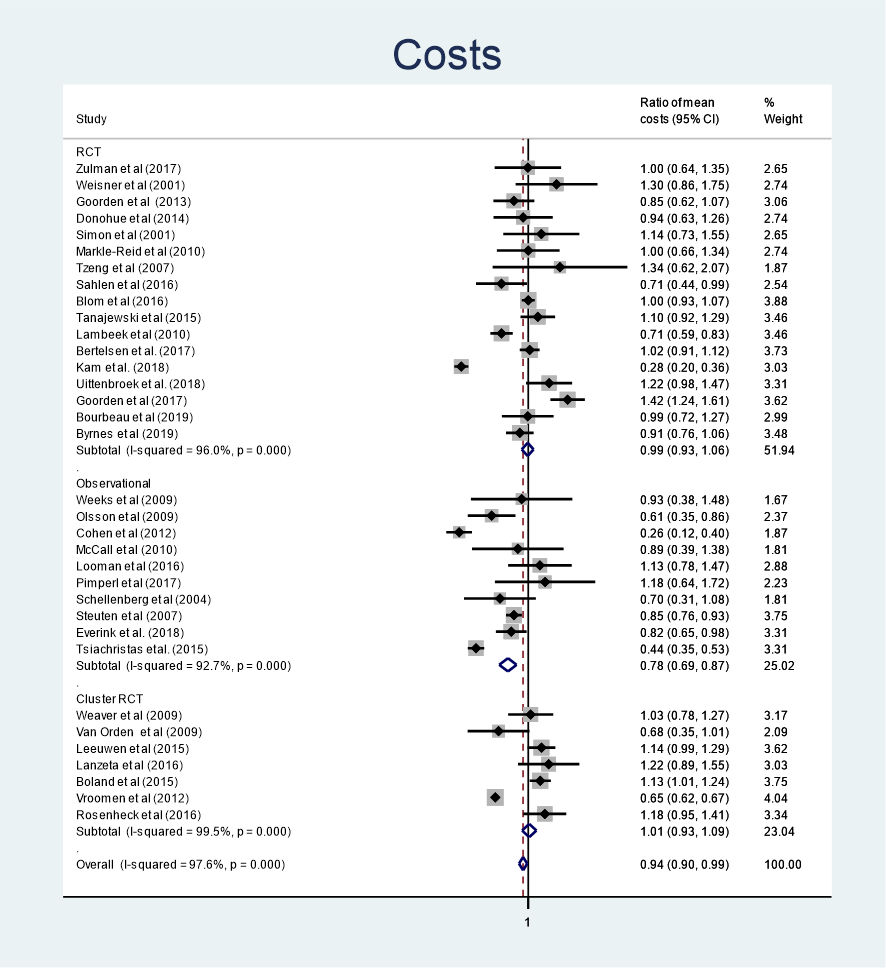
**

2(b)

2(c)

2(e)

**Figure S3: Ratio of mean outcome by (a) region (b) study design (c) study duration (d) type of integrated care intervention**

3(a)

3(b)

3(c)

3(d)
